# ILLNESS PERCEPTION AS A DETERMINANT OF SELF-RATED HEALTH AMONG OLDER CHINESE AMERICANS WITH TYPE 2 DIABETES

**DOI:** 10.1093/geroni/igz038.1829

**Published:** 2019-11-08

**Authors:** Ya-Ching A Huang, Alexandra Garcia

**Affiliations:** 1 Texas State University, Round Rock, Texas, United States; 2 University of Texas at Austin, Austin, Texas, United States

## Abstract

Self-Rated Health (SRH) has been used as a proxy to evaluate individuals’ quality of life, overall well-being, and mortality. However, little is known about how illness perception influences SRH in Chinese American patients with type 2 diabetes (T2DM). This study, guided by Leventhal’s Self-Regulatory Model, explored the association between illness perception and SRH beyond socioeconomic and health factors. A cross-sectional survey from 109 community-dwelling foreign-born Chinese Americans with T2DM (60-95 years old; 51.4% females; Mean age= 74.17, SD=6.83). In addition to descriptive and correlation analysis, hierarchical regression models of SRH was estimated by subsequently entering the following set of predictors: (1) demographics (age, gender, marital status, education, financial status, and acculturation), (2) health factors (insulin usage, length of diabetes, number of chronic condition, depression, and diabetes distress) and (3) illness perception (timeline, disease consequence, personal and treatment control). SRH was measured by asking “How would you rate your overall health?” with a likert response scale: 1 (poor) to 4 (very good). Only 50.5 % of participants rated their health as good or very good. Participants with lower acculturation, higher number of chronic health conditions, higher diabetes distress, and a belief that illness will have more negative consequences were highly correlated with worse SRH. The total amount of variance explained by the entire model was 56% (F = 6.07, p < .001). These findings suggest that the modifiable factors such as acculturation, diabetes distress, and the belief of diabetes consequences should be incorporated into integrative health promotion efforts for this population.

